# Persistent defensive reactivity during extensive avoidance training as a potential mechanism for the perpetuation of safety behaviors

**DOI:** 10.1038/s41598-024-76175-6

**Published:** 2024-10-29

**Authors:** Joscha Franke, Christiane A. Melzig, Christoph Benke

**Affiliations:** 1 Department of Clinical Psychology, Experimental Psychopathology and Psychotherapy, Institute of Psychology, Philipps University Marburg, Marburg, Germany; 2 Center for Mind, Brain and Behavior (CMBB), University of Marburg and Justus Liebig University, Giessen, Germany; 3 Department of Clinical Psychology, Experimental Psychopathology and Psychotherapy, Institute of Psychology, Philipps University Marburg, Gutenbergstraße 18, Marburg, D-35037 Germany

**Keywords:** Exposure therapy, Escape, Fear conditioning, Defensive behaviors, Human behaviour, Anxiety

## Abstract

Safety behaviors are core features of anxiety-related disorders, specifically involving behaviors that do not completely terminate aversive situations, but rather prevent the risk of the occurrence of the expected aversive outcome (US). This study aimed to examine the dynamics of defensive reactivity associated with safety behaviors, both before and after their execution, and to investigate changes in these reactions following extensive training. Twenty-four healthy participants underwent a US-avoidance task as an experimental analog to model safety behavior, requiring a button press to avoid the US without terminating the associated warning cue (CS). Two CS+ were used, with one being devalued during a devaluation procedure to assess the formation of avoidance habits. Defensive reactivity was assessed using fear-potentiated startle (FPS) and skin conductance responses (SCR). Participants showed pronounced defensive reactivity to both CS + vs. CS- before the avoidance action, which decreased upon the opportunity to avoid the US. Contrarily, after the avoidance action, a re-emergence of defensive responses was observed as indicated by FPS and increased SCR. Only one participant showed signs of avoidance habit formation. The findings reveal a re-emergence of defensive reactivity following safety behaviors challenging the prevailing belief that relief is the primary sustaining factor of safety behavior.

**Supplementary Information:**

The online version contains supplementary material available at 10.1038/s41598-024-76175-6.

## Introduction

When confronted with stimuli or situations that are unpleasant, harmful or threatening, individuals tend to engage in avoidance behaviors in order to create an actual or at least psychological distance. Although such behaviors may be adaptive as a short-term coping strategy, excessive or maladaptive avoidance behaviors can perpetuate anxiety and impair daily functioning in the long run^1,2^. In fact, avoidance has been identified as a core element of many mental disorders, such as anxiety disorders, obsessive-compulsive disorder, and post-traumatic stress disorder^3–5^. From a clinical perspective, avoidance behaviors may manifest through actions aiming to achieve the evasion of anxiety-eliciting stimuli or situations^3,6^. For example, individuals with social anxiety disorder might completely avoid attending a social event in order to prevent the occurrence of the individual’s central concern (e.g., being ostracized). Instead of avoiding such aversive social situations, individuals might instead face the feared situation (e.g., attending a social event) while engaging in behaviors that do not completely terminate the situation (e.g., speaking little or quietly, avoiding eye contact), but rather minimize or prevent the risk of the occurrence of the expected aversive outcome (e.g., social rejection). Avoidance behaviors are highly prevalent in anxiety disorders and are thought to impede the successful extinction of learned fears, thereby contributing to the maintenance of pathological anxiety^5–8^.

In human experimental studies, this avoidance behavior is operationalized as a behavior (e.g., a button press) that prevents the delivery of a threat (i.e., the unconditioned stimulus; US) without terminating the threat-signaling cue (i.e., the conditioned stimulus, CS+). In experimental terminology, this type of avoidance is referred to as US-avoidance, which is the most commonly studied type of defensive behavior in humans (Pittig et al.,^9^). In contrast to human experimental studies, rodent studies focus primarily on CS escape, where the response is to shuttle to a safe context, terminating the CS and avoiding the US^2,10^. In clinical contexts, for example, these behaviors reflect situations in which patients escape anxiety-provoking situation, such as abruptly ending social interactions or quickly leaving a crowded place like a supermarket or a bus (Pittig et al.,^9^).

Previous findings from human and animal studies revealed that exerting control over a threat, either through US-avoidance or CS-escape, leads to suppression of defensive reactions to the CS^9,11–14^. For example, studies in rodents demonstrated that fear-induced freezing in response to threat-signaling cues gradually declines as rodents learn to avoid the delivery of the US by shuttling to a safe context^10,15–17^. It has also been demonstrated that this decrease in defensive reactions (i.e., freezing) in favor of defensive actions involves a switch in amygdala pathways, governed by the prefrontal cortex which suppresses central amygdala mediated expressions of defensive reactions and thus facilitates aversively motivated actions^3,18–21^. With extensive training or overtraining, these more goal-directed actions are assumed to become habits - an observation dating back to the beginning of avoidance research (Solomon et al.,^22^). Initial evidence in rodents emphasizes a dorsal striatum-dominated circuitry, that is independent of the amygdala, and mediates these defensive habits^2,18,21^. Similar to rodent models, preliminary evidence in humans indicates analogous changes in brain’s circuit activation in response to the CS+ during US-avoidance training (Delgado et al.,^12^), which is in line with findings showing a reduction of conditioned skin conductance responses to the CS+ in the course of US-avoidance^11–14^.

While these previous findings have enhanced our understanding of the neurobiological mechanisms and circuits involved in avoidance behaviors, their translation to US-avoidance behavior is limited as they predominantly employed CS escape paradigms or focused on defensive reactivity to the CS occurring prior to the initiation of an avoidance response^10,11,13–18,20,21^. However, unlike these paradigms, US-avoidance behavior does not entirely prevent or terminate the CS, that is, individuals are still exposed to the fear-inducing stimulus (Pittig et al.,^9^). Moreover, in these previous studies, a full characterization of defensive responses prior to and after the execution of US-avoidance behavior is lacking. These are, indeed, crucial factors, considering that current perspectives on the maintenance of avoidance behavior diverge, especially regarding the responses in the aftermath of engagement in avoidance behavior. For example, it is proposed that avoidance behaviors may elicit a relief response and diminish fear and anxiety^8,23–25^, which is suggested to perpetuate such behaviors (Mowrer,^26^). Conversely, the uncertainty following US-avoidance behavior may lead to doubts about the effectiveness of the actions performed in avoiding the aversive outcomes^3,9^, possibly resulting in persistent apprehension and repeated execution of avoidance behaviors – which might spiral into a vicious circle contributing to the persistence of excessive US-avoidance behavior.

So far, the dynamics of defensive reactivity during US-avoidance behavior remain unclear, particularly in the aftermath of avoidance when the CS is still present. Therefore, this study aims to close this gap by characterizing defensive reactivity both before and after US-avoidance behavior for elucidating the mechanisms underpinning the maintenance of this behavior. To model a real-world scenario, where perfect control over a situation can rarely be achieved and individuals are uncertain about the effectiveness of their avoidance behavior in preventing exposure to their central concerns, we introduced unpredictability by setting the effectiveness of the avoidance response at 80%, using a partial reinforcement schedule^27–32^. Moreover, earlier research into human avoidance behavior predominantly relied on skin conductance responses as an indicator of fear, despite the fact that skin conductance responses are not specific to fear but rather reflect phasic increases in sympathetic arousal and orienting responses (Lang et al.,^33^). For a comprehensive characterization of defensive activation, in the present study, in addition to conditioned skin conductance responses, we used a translational indicator of freezing behavior, that is the potentiation of the startle response which is mediated by the defensive circuits centered on the amygdala^11,16,34–37^. In line with previous studies^11,13,14,38,39^, in the present study, we expected that defensive reactivity to the CS+ prior to the avoidance by button press would decrease over the course of repeated trials – aligning with findings that perceived control over threat and even the intention to perform avoidance behavior can reduce anxiety^13,40,41^). The period after the avoidance behavior can be conceptualized as a period of anticipation where individuals are uncertain about the effectiveness of their response in preventing the aversive outcome. Previous studies have demonstrated that startle potentiation and heightened SCR are observed in response to threatening stimuli and in anticipation of aversive feedback or threats^42–45^, with particularly elevated responses in situations involving high unpredictability of threat^46–49^. Therefore, after the button press, due to the unpredictability of its success, we assumed that defensive reactivity will remain pronounced over the course of repeated trials. Conversely, and in contrast to studies where the CS is terminated and clear feedback confirms that the aversive outcome was successfully avoided^25,39,50^, we did not expect the execution of the avoidance response to lead to an immediate relief response which would manifest in an attenuated startle response and increased SCR. Such a response pattern is typically observed during relief-associated, pleasant, or rewarding stimuli^25,39,51–53^.

Moreover, we aimed to explore how defensive reactions evolve after extensive training (i.e., after 30 presentations of the CS+), particularly when US-avoidance behavior may have become habitual and thus insensitive to outcome value, as previously observed in both animal and initial human studies^7,9,21,54,55^. Some studies with clinical and non-clinical populations have proposed that 30 repetitions of the CS+ may be sufficient to induce overlearning and habit formation^56–58^. To investigate the formation of habits, we adapted a devaluation procedure used in previous studies (Gillan, Otto, et al.,^59^) in which one CS+ was associated with avoidance of an aversive outcome (i.e., a unpleasant electric shock) by a button press using the left hand, while the other CS+ involved avoidance by a button press with the right hand. To test for the formation of habits, one of the CS+ was devaluated by disconnecting the shock electrode from the shocker device after extensive US-avoidance training. In the present study, we hypothesized that extensive US-avoidance training would lead to the formation of avoidance habits, as evidenced by participants continuing to press the button despite the disconnection of the shock electrode from the shocker device. Moreover, through overtraining, the goal directed component of avoidance actions can become obsolete and the actions can become habits, which is expected to result into diminished differential responding to CS+ and CS-^2,9,55^.

## Methods

### Participants

The sample size in the present study was based on previous studies in the area of fear conditioning and avoidance learning^25,32,37,48^. These previous studies indicated that our study should be sufficiently powered to detect a medium effect size. In our a-priori sample size calculation, a sample size of 19 participants was calculated to detect a medium effect (f^2^ = 0.25) and to achieve a power of 80% for a repeated measures model with an alpha level of 0.05. We expected that 20% of the participants would drop out during the experiment due to potential problems during physiological recordings. Therefore, we included 24 healthy individuals (*n* = 19 female) aged between 18 and 30 years (*M* = 22.25, *SD* = 2.92) in the described study. In order to be included in the study, a standardized interview via telephone was conducted to screen for the following exclusion criteria: (a) severe cardiovascular, respiratory, and neurological diseases (for example a stroke, epilepsy, multiple sclerosis), (b) current or past psychological treatment, (c) impaired hearing or sight, and (d) pregnancy. Participants signed the informed consent and received financial compensation or course credits for their participation. For further characterization of the sample, we assessed trait anxiety via the trait portion of the State-Trait-Anxiety-Inventory (STAI;^60,61^), intolerance of uncertainty via the Intolerance of Uncertainty Scale (IUS; Freeston et al.,^62^), and anxiety sensitivity using the Anxiety Sensitivity Index-3 (ASI-3; Taylor et al.,^63^). The description of sample characteristics is presented in Table 1. The study protocol was approved by the ethics committee of the German Psychological Society. All methods were performed in accordance with the relevant guidelines and regulations.

**Table 1:** Descriptive demographic characteristics and self-report measures.

	M	SD	Range
**Gender (female**,** %)**	79.17		
**Age**	22.23	2.92	18–30
**Anxiety Sensitivity Index (ASI-3)**	22.04	12.20	8–48
**Intolerance of Uncertainty Scale (IUS)**	59.44	17.47	33–101
**State-Trait Anxiety Inventory (STAI)**	35.96	10.35	23–58

Note. M = mean; SD = standard deviation.

### Stimuli and materials

#### Startle probes

To elicit startle responses, a 50 ms burst of broadband white noise with a fall/rise time of < 1 ms was presented binaurally with an intensity of 95 dB through AKG K-66 headphones.

#### Conditioned stimuli and inter-trial interval

Three geometric shapes (triangle, square, circle) with different colors (blue, orange and yellow) that were surrounded by a white frame served as conditioned stimuli (CS). A white cross in the center of a black background projected onto a 1.50 × 1.30 m screen in front of the participants served as inter-trial interval (ITI).

#### Unconditioned stimuli

An electrotactile stimulation delivered to the right or left forearm served as the unconditioned stimulus (US). The electrotactile stimulation occurred for a period of 625 ms and consisted of 125 single pulses each with a duration of 2 ms and a 3 ms break between pulses. Shock electrodes (E.SB010, Digitimer Ltd., UK) were attached over the Nervus Medianus of the left and right arm. The electrical stimulation to the right forearm was delivered by a S48 stimulator including a constant current unit and a subject isolation unit (Grass Instruments, USA), while a DS7A constant current stimulator (Digitimer Ltd., UK) was used for delivery of the electrical stimulation to the left forearm. The intensity of the electrotactile stimulation was adjusted for the right and left side individually to be uncomfortable but not painful. Stimulation intensity did not differ between the left (*M* = 1.77, *SD* = 0.84) and right (*M* = 1.86, *SD* = 0.77) forearm, *t*(21) = 0.333, *p* = .742.

#### Subjective ratings

Participants rated the intensity of their anxiety during CS presentation on a 10-point Likert scale from 1 (*no anxiety*) to 10 (*maximal anxiety*). Participants also rated the likelihood of receiving an electrotactile stimulation during presentation of geometric shapes from 0 to 100%. Ratings were obtained after the acquisition and avoidance phase, after the devaluation instruction including the removal of the shock electrode and after the devaluation phase.

### Physiological recordings

#### Electromyographic activity (EMG)

Electromyographic (EMG) activity was recorded to measure the eye blink component of the startle response. Two electrolyte-filled (GE Medical Systems Information Technologies) Ag/AgCl miniature surface electrodes (3 mm, Sensormedic, Yorba Linda, CA) were placed under the lower eyelid on the left orbicularis oculi muscle. A Coulbourne S75-01 amplifier with a 30-Hz high-pass filter and a Kemo KEM-VBF8-03 400-Hz low-pass filter were used to amplify the raw EMG signal. Digital sampling was performed with a 12-bit A/D converter starting 100 ms before the onset of the startle stimulus and lasting 400 ms following the startle probe with a rate of 1000 Hz.

#### Electrodermal Activity (EDA)

Electrodermal activity (EDA) was recorded from the hypothenar eminence on the palm of the participant’s non-dominant hand. Two silver-silver chloride standard electrodes (8 diameter, Marquette Hellige) that were filled with a 0.05 M sodium chloride electrolyte medium were used. A constant DC voltage of 0.5 V was applied across electrodes (attached 15 mm apart) by a Coulbourn S71-22 skin conductance coupler that processed the signal with a resolution of 0.01 µS. The DC voltage amplified signal was continuously sampled at 10 Hz by a 12-bit A/D converter.

### Procedure

After checking the quality of the signals, the experimental procedure started with a *shock-workup*, during which the intensity of the electrotactile stimulation was adjusted separately for the right and left arm. Starting with a stimulation of 1 mA, the intensity was varied until the participants rated the level of electrical stimulation as unpleasant but not painful.

**Figure 1:**
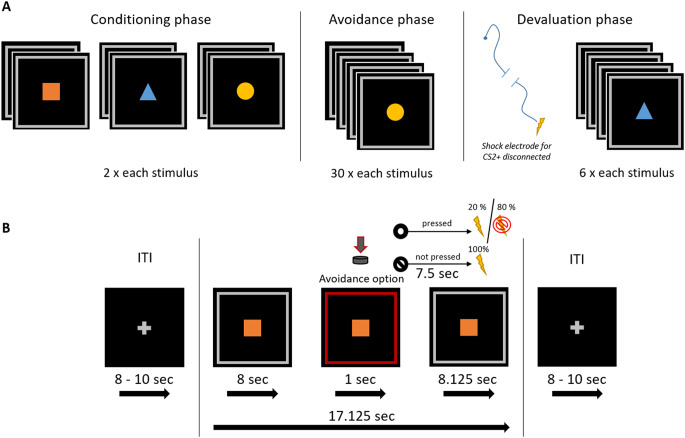
Illustration of the experimental design. Note. Trial timeline and experimental design. Panel **A**: Procedure of the experiment consisting of a conditioning, avoidance and devaluation phase, each featuring 3 stimuli with differing trial numbers per phase. Panel **B**: Time course of a prototypical trial from the avoidance phase.

The experimental design is shown in Fig. 1. The experiment began with a *habituation phase* in which 6 startle probes were presented. In the *instructed fear conditioning phase* (C), the subjects were instructed that three different geometric figures in differing colors would be presented. Moreover, participants were told that one figure signaled an electrotactile stimulation to the right arm, another one signaled stimulation to the left arm, and the third one signaled the absence of stimulation. Thus, participants were explicitly instructed about the contingencies between the CSs and US. To ensure participants understood the instructions correctly, they were asked to repeat the information about the contingencies. As the research question of this study pertains to the avoidance phase, we employed an instructed fear acquisition procedure to ensure that all participants were aware of the contingencies and exhibited a reliable conditioned fear response to the CSs before the avoidance phase (see Gillan et al.^57^) for a similar study design). In line with previous studies showing rapid fear response formation following instructed acquisition procedures each stimulus was presented twice during this first phase^57,64–66^. The CSs were presented for 17.125 s. Startle probes were presented between 5 and 7 s and between 14 and 16 s after CS onset in all trials (3 different CS shown 2 times each and 2 startle probes per stimulus led to a total of 12 startle stimuli in this phase). The electrotactile stimulation was delivered 16.5 s after CS onset. Each symbol was presented two times in pseudo-randomized order. ITIs between CSs lasted between 8 and 10 s. Two startle probes were presented 5 to 7 s after onset of the ITIs. At the end of the phase, participants rated the intensity of experienced anxiety and the likelihood of receiving an electrotactile stimulation during presentation of the geometric shapes.

In the following *avoidance phase* (A), stimuli duration and timing during a trial remained the same. Participants were instructed that the same CSs as in the acquisition phase would be presented, but they could prevent the delivery of the electrotactile stimulation by quickly pressing a button when the white frame around the geometric figure turned red. The red frame was presented for 1 s starting at 8 s after the CS onset. Participants were instructed that the stimulation on the right or left arm can be prevented by pressing the button with the respective hand. Participants were instructed that late or incorrect (wrong sided) button presses during a CS+ resulted in the delivery of the electric stimulation. A partial reinforcement rate of 80% was used in that button presses were only partially effective in cancelling the shock (i.e., in 80% of the trials). That is, even if participants pressed the button correctly, USs were delivered in 20% of trials (US reinforcement rate: 20%), while the US was delivered whenever participants did not press the button during the CS+ (US reinforcement rate: 100%). This reinforcement rate was not disclosed to the participants during the instruction. A CS- was never followed by the US. The symbol was presented for 8.125 s after the presentation of the red frame. Again, startle probes were delivered between 5 and 7 s and between 14 and 16 s after CS onset in each trial. The timing of the startle probes was specifically chosen to be presented both before (i.e. 5 to 7 s after CS onset) and after (i.e. 14 to 16 s after CS onset) the button press option. Additionally, startle probes were presented 5 to 7 s after onset of the ITI in 20% of the ITIs (18 times). Each CS was presented 30 times in counterbalanced order. At the end of the phase, participants were again asked to rate the intensity of their anxiety and the probability of receiving a stimulation for each CS.

During the last experimental phase, *devaluation with shock removal* (D), the experimenter disconnected one shock electrode from the shock stimulator in a manner that was clearly visible for the participants. Participants were instructed that they could no longer receive stimulation following the presentation of the corresponding symbol (CS2+), while the shock electrode attached to the other arm remained connected to the shock stimulator and the stimulation could still be applied following presentation of the other stimulus (CS1+). The arm on which the attached electrode was disconnected from the stimulator was counterbalanced between participants. After disconnecting one electrode from the stimulator, participants were asked to rate their anxiety and the probability of receiving electrotactile stimulation. Prior to the start of the last phase, participants were instructed that it was still possible to avoid stimulation by pressing a button, similar to the previous phase. The removal of the electrode, instructions, and participants’ ratings between the avoidance and devaluation phase lasted approximately 2 min. Each geometric figure was presented 6 times in pseudorandomized order. The timing for the startle stimuli, electrotactile stimulation, and ITI, were equivalent to the previous phases. A startle probe was delivered in 3 of the ITIs. Afterwards, participants once more rated the intensity of their anxiety and the probability to receive stimulation. After the experiment, participants completed a set of questionnaires.

### Data reduction

A 60-Hz high-pass filter was applied offline to the digitalized raw EMG signal. The signal was then rectified and smoothed using a first-order low-pass filter with a time constant of 10 ms. The startle eyeblinks were scored semi-automatically using a computer program (Globisch et al.,^67^) that identified blink onset (20–100 ms after probe delivery) and peak amplitude (within 150 ms after probe delivery). Each individual startle eyeblink response was checked and corrected manually if necessary. Only trials in which blinks started between 20 and 100 ms after the startle probe was delivered and reached their peak amplitude within 150 ms were scored as valid startle responses. If no blink was detected within the defined time window, the trials were scored as zero responses. In accordance with the guidelines for human startle eyeblink studies (Blumenthal et al.,^68^), trials were rejected (5.6%) and treated as missing if there was excessive noise due to technical problems, spontaneous eye-blinks or movement artefacts. Digital values were converted to microvolts (µV) and then exported. To correct for the inter-individual variability not related to the experimental manipulation, all values were standardized by z-transformation and then converted to T-scores (M = 50, SD = 10) as recommended by the guidelines for human startle eyeblink studies (Blumenthal et al.,^68^).

The digital values of electrodermal activity were converted to microsiemens (µS). The skin conductance response (SCR) for each trial was calculated as the difference between a baseline and the maximum value in the interval of 0.5 and 5 s after the event of interest. The baseline was defined as the mean of 1 s before the presentation of the stimulus. Trials were treated as missing if there was excessive noise due to technical problems or movement artefacts. If missing values were present during the baseline period, the SCR was set to not a number (NAN). If no reaction could be found in the interval of interest, a zero response was determined. Finally, the values were logarithmized and range corrected according to Lykken and Venables^69^. The data of two participants had to be excluded due to loss of the signal during the experiment or excessive noise in the data (e.g., when the participants moved excessively).

Only one participant continued to press the avoidance button for the devaluated stimulus (CS2+). This could be a sign that the person developed habits. Since it was only one participant who might have developed an avoidance habit, we were unable to analyze the effect of habit formation on defensive reactivity. As responses from this participant might deviate from other participants’ responses due to habit formation, we decided to exclude this participant from all analyses.

### Data analysis

For the analysis, trials were averaged as blocks per stimulus to analyze the SCR and the fear potentiated startle response (FPS) throughout the experiment. For the conditioning phase, all trials were averaged per CS or ITI. In the avoidance training phase, 5 trials were averaged, and in the devaluation phase, 2 trials per block were averaged. For the analysis, the CS2 + is the stimulus for which the electrode was removed in the last phase of the experiment, so an electrotactile stimulation could be received for CS1 + whenever no avoidance response was made or possible. SCR and FPS were analyzed separately before and after the time window in which an avoidance reaction could be performed. Successful fear acquisition was ascertained by applying a Type III repeated-measures analysis of variance (ANOVA) with the within-subjects factor *stimulus* (CS1 + vs. CS2 + vs. CS- vs. ITI). To examine changes in SCR and FPS to CSs over the course of avoidance and devaluation, an ANOVA was applied with *stimulus* and *block* (block 1 to block 6 for avoidance training, block 1 to block 3 for devaluation phase) as within-subjects factors. Self-reported fear and probability of receiving a shock were analyzed with an ANOVA with *phase* (after conditioning, after avoidance, after disconnecting shock electrode from shocker, and after devaluation) and *stimulus* (CS1 + vs. CS2 + vs. CS-) as within-subjects factors. A *Greenhouse-Geisser* correction was applied when necessary. A significance level of *p* < .05 was used. In addition to null hypothesis significance testing, we calculated Bayes Factors (BF). A Bayesian approach allows for gathering evidence in favor of the alternative hypothesis as well as the null hypothesis, or relative evidence when comparing statistical models. We report *BF*_01_, which indicates the evidence in favor of the null hypothesis, or *BF*_10_, which indicates the evidence in favor of the alternative hypothesis. For example, a *BF*_10_ of 5 would indicate that the data are 5 times more likely to occur under the alternative hypothesis than under the null hypothesis. For the Bayesian analyses separate tests were used for ANOVAs and t-tests, as described in previous avoidance conditioning studies^70–72^. Due to insufficient information about the prior effect size distribution as well as uncertainties about the actual benefit of using subjective priors, we conducted the analyses with the default Cauchy (*r* = .707) for the alternative hypothesis^70,73,74^.All analyses were performed in R (version 4.2.2 R Core Team,^75^). Additional packages that were used included *dplyr* (Wickham et al.,^76^) *reshape* (Wickham,^77^) for data processing and editing, *ggplot2* for the creation of plots and figures (Wickham,^78^), *afex* (Singmann et al.,^79^), *psych* (William Revelle,^80^) and *emmeans* (Lenth,^81^) for the analysis. Bayesian analyses were run with the *BayesFactor* package (Morey & Rouder,^82^). The power analysis was performed in G*Power 3.1.9.7. (Faul et al.,^83^).

## Results

### Instructed fear conditioning phase

**Figure 2:**
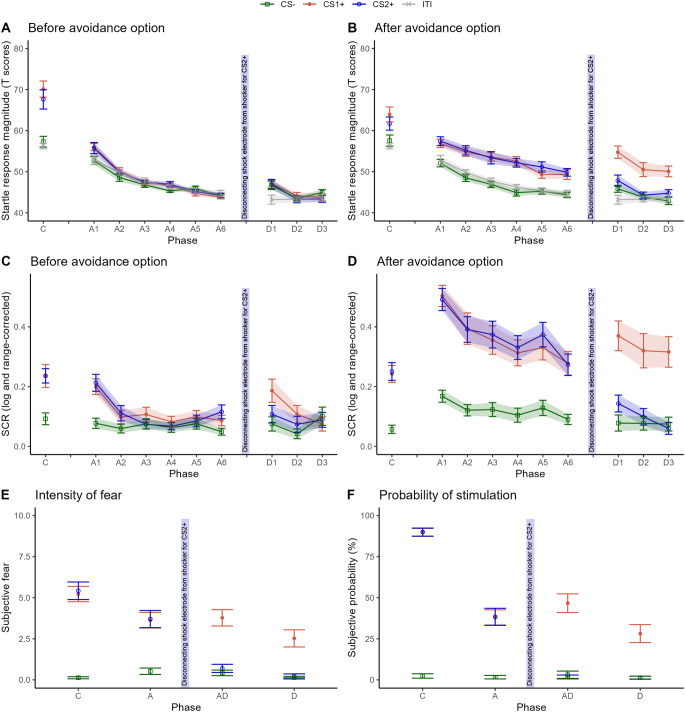
Subjective measures, skin conductance response and startle responses for conditioning, avoidance and devaluation phase. Note. Means and standard errors for the subjective measures (bottom panel), skin conductance response (middle panel), and startle responses (top panel). X-axis show the blocks for each phase (C: fear acquisition / conditioning; A: avoidance phase; AD: after disconnecting shock electrode from shocker for CS2+; D: devaluation phase).

During the acquisition phase, all participants showed a strong startle potentiation to both CS+ compared to ITI, while there was no difference between startle response magnitudes to CS- and ITI (Fig. 2A, B). This pattern was observed at early, stimulus *F*(2.06, 41.15) = 20.02, *p* < .001, *η*_*G*_^*2*^ = 0.405, *BF*_10_ > 1000, and late, stimulus, *F*(2.64, 52.78) = 7.10, *p* < .001, *η*_*G*_^*2*^ = 0.177, *BF*_10_ = 135.92, time points during the CS presentation (timing corresponds to phases before and after button presses in the following avoidance learning phase). Post-hoc contrasts confirmed this pattern, early: CS+ s to ITI all *t*s(20) > 5.18, all *p*s < 0.001, all *d*s > 3.28, 95% CI [2.18, 5.86], all *BF*_10_ > 100, CS- to ITI *t*(20) = 0.96, *p* = .351, *d* = 0.54, 95% CI [0.08, 1.00], *BF*_01_ = 2.93, late: CS+ s to ITI all *t*s(20) > 3.56, all *p*s < 0.01, all *d*s > 2.13, 95% CI [1.35, 3.41], all *BF*_10_ > 20, CS- to ITI *t*(20) = 1.21, *p* = .239, *d* = 0.69, 95% CI [0.21, 1.17], *BF*_01_ = 2.30. Moreover, SCR (Fig. 2C, D) was higher for both CS+ than for CS- at early, stimulus *F*(1.68, 35.25) = 12.71, *p* < .001, *η*_*G*_^*2*^ = 0.212, *BF*_10_ = 613.22, and late phases during CS presentation, stimulus *F*(1.94, 40.73) = 20.32, *p* < .001, *η*_*G*_^*2*^ = 0.375, *BF*_10_ > 1000. Again, contrast confirmed these differences (before and after: CS+ s to CS- all *t*s(21) > 3.86, all *p*s < 0.001, all *d*s > 0.34, 95% CI [-0.08, 0.94], all *BF*_10_ > 38.83, CS1+ and CS2+ all *t*s(20) < 0.23, all *p*s > 0.823, all *d*s < 0.02, 95% CI [-0.44, 0.42], all *BF*_01_ < 0.22). After the acquisition, participants reported more fear, *t*s(22) > 10.26, *p*s < 0.001, *d*s > 3.46, 95% CI [2.29, 4.56], *BF*_10_ > 1000, and rated shock delivery as more likely during presentation of both CS+ compared to CS-, *t*s(22) > 28.07, *p*s < 0.001, *d*s > 22.62, 95% CI [15.92, 29.43], *BF*_10_ > 1000 (Fig. 2E, F). Participants did not differentiate between the two CS+ in reported fear, *t*(22) = 0.83, *p* = .415, *d* = − 0.19, 95% CI [-0.60, 0.22], *BF*_01_ = 3.35, or probability of shock, *t*(22) > 0.27, *p* = .793, *d* = − 0.10, 95% CI [-0.51, 0.31], *BF*_01_ = 4.43.

### Avoidance phase

As depicted in Table 2, nearly all CS+ trials were avoided when the participants were given the opportunity to do so during the avoidance phase (99.14%). A small percentage of CS- trials were also avoided (3.3%).

**Table 2:** Proportion of avoidance reactions during the last two phases of the experiment.

	CS	%	SD
**Avoidance phase**	CS1+	98.99	0.025
	CS2+	99.28	0.017
	CS-	3.33	0.052
**Devaluation phase (shock removal CS2+)**	CS1+	99.28	0.035
	CS2+	0.00	0.000
	CS-	0.00	0.000

Note. % = proportion of avoided trials; SD = standard deviation.

#### Response before avoidance option

We did not find a main effect of stimulus, *F*(2.05, 41.09) = 1.52, *p* = .230, *η*_*G*_^2^ = 0.013, *BF*_01_ = 29.22, or an interaction between Time and Stimulus, *F*(7.30, 145.92) = 1.57, *p* = .145, *η*_*G*_^2^ = 0.035, *BF*_01_ = 15.94. However, on a trial by trial basis (see Fig. S1), the first trial of the avoidance phase showed a potentiation for CS1+, *t*(21) = 2.67, *p* < .05, *d* = 2.34, 95% CI [1.52, 3.16], *BF*_10_ = 3.66, and CS2+, *t*(21) = 2.59, *p* < .05, *d* = 2.29, 95% CI [1.48, 3.10], *BF*_10_ = 3.19, vs. ITI. There was no potentiation of startle responses to CS- vs. ITI, *t*(21) = 1.47, *p* = .157, *d* = 1.12, 95% CI [0.59, 1.66], *BF*_01_ = 1.76. The potentiation of CS1+ und CS2+ to ITI diminished quickly and was not significant from the second trial on, CS1+, *t*(19) = 1.66, *p* = .114, *d* = -1.10, 95% CI [-1.66, − 0.54], *BF*_01_ = 1.35, and CS2+, *t*(19) = 0.78, *p* = .446, *d* = 0.59, 95% CI [0.12, 1.07], *BF*_01_ = 3.29. For SCR, the amplitudes were greater for CS1+, *t*(21) = 4.91, *p* < .001, *d = 0.36*, 95% CI [-0.07, 0.79], *BF*_10_ = 356.77, and CS2+ than for CS-, *t*(21) = 5.84, *p* < .001, *d* = 0.41, 95% CI [-0.03, 0.85], *BF*_10_ > 1000, during the first block of the avoidance phase. The differentiation was significant in the second block for CS2+ vs. CS-, *t*(21) = 2.10, *p* < .05, *d* = 0.15, 95% CI [-0.27, 0.57], *BF*_10_ = 1.39, but not for CS1+ vs. CS-, *t*(21) = 1.59, *p* = .127, *d* = 0.11, 95% CI [-0.31, 0.54], *BF*_01_ = 1.51. In the following blocks, the difference in SCR between CSs was absent. This pattern was confirmed by a significant Stimulus by Time interaction, *F*(6.88, 144.39) = 4.10, *p* < .001, *η*_*G*_^2^ = 0.05, *BF*_10_ = 23.04.

#### Response after avoidance option

Throughout the avoidance phase, participants showed a strong potentiation of the startle response magnitudes after they pressed the button to avoid shock delivery during CS1+ and CS2+ vs. ITI, which results in a main effect for Stimulus *F*(2.23, 44.54) = 29.92, *p* < .001, *η*_*G*_^2^ = 0.278, *BF*_10_ > 1000. The Stimulus by Time interaction conversely was not significant, *F*(7.34, 146.84) = 0.63, *p* = .741, *η*_*G*_^2^ = 0.013, *BF*_01_ = 412. Contrasts showed differences for all blocks between ITI and CS1+, all *t*s(20) > 2.57, all *p*s < 0.05, all *d*s > 1.49, 95% CI [0.86, 3.44], all *BF*_10_ > 5.46, as well as CS2+, all *t*s(20) > 2.91, all *p*s < 0.01, all *d*s > 1.70, 95% CI [1.02, 3.57], all *BF*_10_ > 5.71, but not between ITI and CS-, all *t*s(20) < 1.57, all *p*s > 0.132, all *d*s < 0.19, 95% CI [-1.19, 0.62], all *BF*_01_ > 1.53. Correspondingly, after button press, SCR amplitudes were also increased during CS1+ and CS2+ compared to CS- (contrasts for both CS+ to CS- over blocks: all *t*s(21) > 4.74, all *p*s < 0.001, all *d*s > 0.43, 95% CI [0.02, 1.34], all *BF*_10_ > 252.44). This differential responding slightly decreased toward the end the of the avoidance phase (interaction Stimulus by Time *F*(6.54, 137.29) = 2.90, *p* < .01, *η*_*G*_^2^ = 0.019, *BF*_01_ = 15.14). At the end of the phase, although fear and shock probability ratings decreased, participants still reported more fear, *t*s(22) > 6.27, *p*s < 0.001, *d*s > 2.03, 95% CI [1.31, 2.76], *BF*_10_ > 1000, and rated the shock as more likely to occur, *t*s(22) > 7.95, *p*s < 0.001, *d*s > 7.80, 95% CI [5.46, 10.59], *BF*_10_ > 1000, during both CS+ as compared to CS- (see Fig. 2, panel E and F).

### Devaluation phase

After the shock electrode was disconnected from stimulator (for CS2+), fear and US-expectancy ratings for CS2+ decreased and no longer differed from ratings of CS-, *t*s(22) < 1.01, *p*s > 0.322, *d*s < 0.15, 95% CI [-0.81, 0.65], *BF*_01_ > 2.89. Fear and US-expectancy ratings for CS1+ (shock delivery during CS1+ was still possible) remained elevated as compared to CS- and CS2+, *t*s(22) > 6.68, *p*s < 0.001, *d*s > 2.15, 95% CI [1.40, 11.53], *BF*_10_ > 1000.

#### Responses before avoidance option

Throughout the devaluation phase, there was no significant modulation of the startle response magnitudes to CSs vs. ITI, as shown by a non-significant effect for Stimulus, *F*(2.60, 52.03) = 2.34, *p* = .092, *η*_*G*_^2^ = 0.029, *BF*_01_ = 3.25, and Stimulus by Time interaction, *F*(3.09, 61.79) = 1.56, *p* = .206, *η*_*G*_^2^ = 0.030, *BF*_01_ = 5.88. Only a significant main effect for Time was found which shows a general decline of the startle response over all CSs, *F*(1.96, 39.22) = 6.19, *p* < .01, *η*_*G*_^2^ = 0.062, *BF*_10_ = 78.87. The SCR amplitudes showed a significant effect for Stimulus, *F*(1.69, 33.76) = 3.96, *p* < .05, *η*_*G*_^2^ = 0.027, *BF*_01_ = 1.43, but no Stimulus by Time interaction, *F*(2.72, 54.43) = 1.68, *p* = .185, *η*_*G*_^2^ = 0.034, *BF*_01_ = 3.86. As observed in Fig. 2, the SCR to the CS1+ was elevated compared to CS- during the first, *t*(20) = 2.23, *p* < .05, *d* = 0.23, 95% CI [-0.20, 0.67], *BF*_10_ = 1.73, and second block, *t*(20) = 2.20, *p* < .05, *d* = 0.18, 95% CI [-0.25, 0.61], *BF*_10_ = 1.63, but not during the third block. There were no differences in SCR between CS2+ and CS-, all *t*s(20) < 1.65, all *p*s > 0.114, all *d*s < 0.11, 95% CI [-0.46, 0.54], all *BF*_01_ > 4.19.

#### Responses after avoidance option

As depicted in Fig. 2, In the first block, CS1+ was potentiated compared to ITI, *t*(20) = 6.50, *p* < .001, *d* = 4.13, 95% CI [2.78, 5.47], *BF*_10_ > 1000, as was the devaluated CS2+, *t*(20) = 2.71, *p* < .05, *d* = 1.65, 95% CI [0.99, 2.32], *BF*_10_ = 3.91, and the CS-, *t*(20) = 2.17, *p* < .05, *d* = 1.11, 95% CI [0.56, 1.66], *BF*_10_ = 1.55. For second and third block of trials, only CS1+ remained potentiated compared to ITI, all *t*s(20) > 3.64, all *p*s < 0.01, all *d*s > 2.31, 95% CI [1.48, 3.34], all *BF*_10_ > 23.65, CS2+ and CS- to ITI: all *t*s(20) < 1.28, all *p*s > 0.216, all *d*s < 0.58, 95% CI [-0.75, 1.04], all *BF*_01_ > 2.16. This pattern is substantiated by a significant Stimulus effect, *F*(1.84, 36.83) = 23.33, *p* < .001, *η*_*G*_^2^ = 0.308, *BF*_10_ > 1000, and a non-significant Stimulus by Time interaction, *F*(4.44, 88.81) = 1.61, *p* = .174, *η*_*G*_^2^ = 0.027, *BF*_01_ = 8.02. The SCR to CS1+ was increased compared to CS2+ and CS- across the devaluation phase (stimulus *F*(1.16, 23.11) = 25.25, *p* < .001, *η*_*G*_^2^ = 0.326, *BF*_10_ > 1000, post-hoc: all *t*s(20) > 4.12, all *p*s < 0.001, all *d*s > 0.45, 95% CI [0.01, 1.02], all *BF*_10_ > 62.66). Between CS2+ and CS- no differentiation in SCR was found, all *t*s(20) < 1.61, all *p*s > 0.124, all *d*s < 0.15, 95% CI [-0.47, 0.58], all *BF*_01_ > 1.46. After the devaluation phase, subjective fear and US-expectancy ratings remained elevated only for CS1+ compared to CS- and CS2+, all *t*s(22) > 4.70, all *p*s(22) < 0.001, all *d*s > 1.53, 95% CI [0.92, 6.93], all *BF*_10_ > 252.76.

## Discussion

The present study aimed to elucidate the dynamics of defensive reactivity to repeated execution of US-avoidance behavior. The present study utilized an US-avoidance task where participants pressed a button to avoid an aversive stimulus (i.e., the US) without terminating the associated warning cue (i.e., the CS), paralleling clinical safety behaviors where actions are taken to avoid threats without leaving the anxiety-inducing situation. For example, individuals with social anxiety attend a social event but engage in safety behaviors like speaking quietly or avoiding eye contact to minimize the risk of social rejection (i.e., the expected aversive outcome). Thus, the present US-avoidance task might be conceptualized as an experimental analog to model safety behavior (see Pittig et al.,^9^ for a detailed discussion of the conceptual classification of US-avoidance as safety behavior). In order to gain a more detailed insight into the dynamics of defensive responding to US-avoidance, we measured participants’ psychophysiological reactions before and after the avoidance option was available, thus offering potential insights into processes involved in the maintenance of safety behavior. Additionally, we examined the possible changes of defensive reactions following excessive US-avoidance training, expecting that individuals will form defensive habits due to overtraining.

All participants demonstrated successful fear learning as indicated by pronounced potentiation of the startle and skin conductance responses along with elevated ratings of fear and US-expectancy to both CS+ vs. CS-. This conditioned fear response to both CS+ attenuated quickly and persistently with the opportunity to avoid the presentation of the US by button press. Once the button press was emitted, a re-emergence of potentiated startle responses and increased skin conductance to both CS+ was observed. However, we found no indications for habit formation due to US-avoidance overtraining.

Our findings regarding participants’ defensive reactivity before the button press are consistent with previous animal and human studies, showing that defensive reactions diminish in favor of defensive actions^13,14,38^. Notably, utilizing startle potentiation as a translational measure of amygdala-mediated freezing extends findings from animal models to humans, overcoming the limitations of previous research which primarily relied on skin conductance responses which impeded direct translation. The observed pattern of decreased startle responses prior to the button press in our study mirrors findings from animal studies, where a reduction in freezing behavior was observed when animals were given the opportunity to avoid a threat^10,15,21,37,84^. The suppression of defensive reactions such as freezing in favor of defensive actions in rodents has been well documented to be regulated by the infralimbic prefrontal cortex which suppresses central amygdala mediated freezing, while threat information is directly processed to the nucleus accumbens supporting defensive actions (LeDoux et al.,^2^). Our finding of a decrease in startle potentiation to the CS+ throughout the US-avoidance training align with this neurobiological evidence. Employing startle responses as a translational measure of freezing behavior effectively bridges the gap between human and animal research, offering a deeper insight into the neurobiological processes that underlie US-avoidance behavior.

Contrary to the reduction of defensive reactivity observed before the avoidance action, we found a pronounced potentiation of the startle responses and increased SCR to CS+ after the button press. This is a robust and well-documented response pattern to threat indicating defensive response mobilization mediated by brain defensive networks with the amygdala being the central hub^33,35,36,42,48,85^. This finding suggests that even after an active attempt to mitigate a perceived threat, the continued presence of a potential danger cue coupled with ambiguity about the success of the avoidance behavior triggers the re-emergence of defensive activation. This response pattern has also been observed in situations where individuals anticipate threats or negative feedback^42,46,86,87^. Importantly, previous studies have shown that the startle eyeblink response can also be potentiated during the anticipation of positive outcomes (Bradley et al.,^88^), though this finding has been inconsistent across studies^45,89^. Therefore, it cannot be ruled out that participants were in a state of expecting a positive outcome (i.e., anticipating that the US would not occur due to the button press), which may have contributed to the observed startle potentiation. Based on previous studies that demonstrated that the anticipation of unpredictable threat leads to increased defensive activation^46,86,90^, it is plausible to assume that the unpredictability of threat occurrence due to the partial reinforcement schedule in the present avoidance phase induced a defensive state characterized by startle potentiation and heightened SCR.

In contrast, it has been demonstrated that stimuli associated with relief as well as pleasant or rewarding stimuli elicit an attenuation of the startle response along with an increased SCR^25,39,51–53^. In the present study, we did not observe an appetitive response pattern indicative of a relief reaction in the aftermath of the emitted avoidance response. Instead, individuals exhibited pronounced defensive mobilization after the execution of an avoidance action when the conditioned stimulus remained present, which is typical for situation in which safety behavior is shown. This contrasts to situations where the omission of the threat is unambiguous, achieved by escaping a CS or US, which ultimately reduces uncertainty and elicits relief, as demonstrated in previous experimental studies^25,39,50^. After safety behavior, uncertainty remains or even increases, and the feeling of control decreases^23,91^, which may lead to greater reliance on safety behaviors. It is reasonable to assume that the re-emergence of defensive response mobilization after initiation of safety behavior might entail further safety behaviors which might set the stage of a vicious circle. In contrast to theories explaining the perpetuation of safety and avoidance behavior with a feeling of relief after performing an avoidance response^8,23,25^, the results in this study did not support the importance of relief as a mechanism necessary for the maintenance of safety behavior. The return of defensive mobilization after the button press was immediate, and the period to feel relieved seems to be rather shortsighted. Instead, perceived control over threat along with the elimination of defensive mobilization in anticipation of the execution of safety behavior as well as the re-emergence of defensive response mobilization are reasonable mechanisms that might contribute to the maintenance of safety behavior. Further studies are warranted to gain greater insights into the exact mechanism perpetuating safety behavior. Although this study did not specifically examine cognitive forms of avoidance, such as worry, research suggests that worry may act as a cognitive avoidance strategy, and it has been shown to activate defensive responding^92–94^. The present experimental design could be used in future research to investigate the role of worry as a cognitive avoidance strategy and its impact on defensive mobilization, particularly in clinical populations where this behavior is more prevalent.

During the devaluation phase, the shock electrode was disconnected from the shock stimulator for one CS+ (i.e., the devalued CS+), while for the other CS+ the shock electrode remained connected to the shock stimulator. Participants no longer pressed the button during the devalued CS+, except for one participant. Thus, only one participant showed signs of avoidance habit formation. After the devaluation of the CS+, defensive response mobilization rapidly diminished as evidenced by a lack of differentiation in startle responses and SCR. For the other CS+, participants still pressed the button, while the responses pattern were comparable to those observed in the preceding avoidance training phase. The devaluation phase of our study provided an opportunity to explore the transition from goal-directed actions to habitual behavior in the context of US-avoidance. However, due to the limited evidence of habit formation in our sample, we were not able to characterize potential intra- and interindividual differences in defensive responding between goal-directed and habitual avoidance.

## Limitations

In the present study, only one participant showed indications for habit formation which did not allow to further characterize the dynamics of conditioned defensive reactivity during the transition from goal-directed to habitual avoidance. Moreover, it impeded our analysis of differential responses between habitual and non-habitual avoiders. While previous research has effectively identified avoidance habits in patients with obsessive-compulsive disorder, the repeated avoidance behavior in our training protocol may not have been adequate to induce overtraining and subsequent habit formation in healthy individuals (Gillan, Otto, et al.,^59^). Furthermore, it is possible that the adapted procedure was not sufficiently sensitive to detect the formation of avoidance habits in healthy individuals^54,55^. Future studies, therefore, might extend the length of avoidance training or might apply a different experimental habit formation test to examine habits and the costs that come with avoidance behaviors^5,55,95^. While startle probes are generally rated as neutral to slightly unpleasant in previous studies (e.g. Neubert et al.,^96^), it is possible that their aversiveness, though lower than the electrotactile stimulation in previous studies, could have influenced participants’ avoidance responses. Previous research has shown that acoustic startle probes can delay the acquisition of fear, potentially affecting avoidance behavior^97,98^. Moreover, a brief 2-minute break occurred between the avoidance and devaluation phases, during which we observed an increase in startle responses and SCR to all CSs before the avoidance option, as well as to CS1+ after the avoidance option. While spontaneous recovery of fear typically occurs over longer intervals (e.g., 24 h;^99–101^), it is possible that spontaneous recovery of fear could have contributed to the increase in responding during this short gap. Alternatively, this increase might be due to sensitization caused by the instructions (e.g., reminders that shocks are still possible) or the removal of the shock electrode for CS2+, which could have highlighted that shock delivery was still possible for CS1+. Furthermore, it should be noted that the experimental tasks could have increased state anxiety, potentially affecting the responses to the questionnaires administered after the experimental task. However, in the present study, we used trait versions of the questionnaires which in theory should not be influenced by state anxiety. In fact, the means and standard deviations of the total scores of the questionnaires in our study are comparable to those reported in previous studies in which questionnaires were delivered prior to experiments^66,102^, suggesting a limited effect of the timing of questionnaire delivery on questionnaire scores. Subjective reports of relief could also be collected to understand whether or not people’s subjective relief response is consistent with their innate psychophysiological state or not. Moreover, the low reinforcement rate during US-avoidance may have increased unpredictability in the present study, which has been demonstrated to heighten startle reactivity, impair the ability to discriminate between CS- and CS+, and to contribute the formation of inflexible avoidance behaviors, particularly in individuals with high intolerance of uncertainty^46–49^. Future studies should ensure sufficient power to detect potential moderating effects of interindividual differences, such as trait anxiety or intolerance of uncertainty, on the dynamics of defensive reactivity during US avoidance in similar experimental paradigms. In addition, the present findings have to be extended to clinical populations in order to investigate potential differences in the formation of habits and dynamics of defensive reactivity to US-avoidance behavior. Furthermore, the use of instructed fear acquisition in this study may have influenced the subsequent avoidance learning phase. Future research should consider replicating the present findings using uninstructed associative fear learning.

## Conclusions

This study aimed to explore the dynamics of defensive reactivity in relation to US-avoidance behaviors. In a clinical context, this form of avoidance is typically referred to as safety behavior, a core feature in various anxiety-related disorders. Our findings revealed that participants demonstrated a marked reduction in defensive reactivity when given the opportunity to avoid an aversive stimulus. However, upon executing the avoidance action, a re-emergence of heightened defensive responses was observed. This challenges the prevailing notion that relief following avoidance actions is the primary mechanism sustaining safety behaviors. Instead, our findings indicate that increased defensive mobilization potentially triggered by the persistent uncertainty and perceived lack of control might play a significant role in maintaining these behaviors. Given the absence of habit formation in our sample, future research should aim to clarify the conditions that lead to habitual avoidance, particularly in clinical populations where these behaviors might be more common. This understanding is crucial for developing targeted interventions to address these deeply rooted avoidance habits.

## Supplementary Information

Supplementary Material 1.

## Data Availability

The data, that support the findings of this study, are available from the corresponding author (christoph.benke@uni-marburg.de) upon reasonable request.
