# Supplementary material for: Persistent defensive reactivity during extensive avoidance training as a potential mechanism for the perpetuation of safety behaviors
Source: Sci Rep. 2024 Oct 29;14:25925. doi: 10.1038/s41598-024-76175-6 (PMC11522625; doi:10.1038/s41598-024-76175-6)
Supplement: Supplementary file 1 — Supplementary Material 1. [file 41598_2024_76175_MOESM1_ESM.docx]

**Supplement**

**Persistent defensive reactivity during extensive avoidance training as a potential mechanism for the perpetuation of safety behaviors**Joscha Franke ^[[1]](#footnote-1)^, Christiane A. Melzig ^1, 2^, Christoph Benke ^1^

**Figure S1.** Trial by trial plots for startle responses and skin conductance response
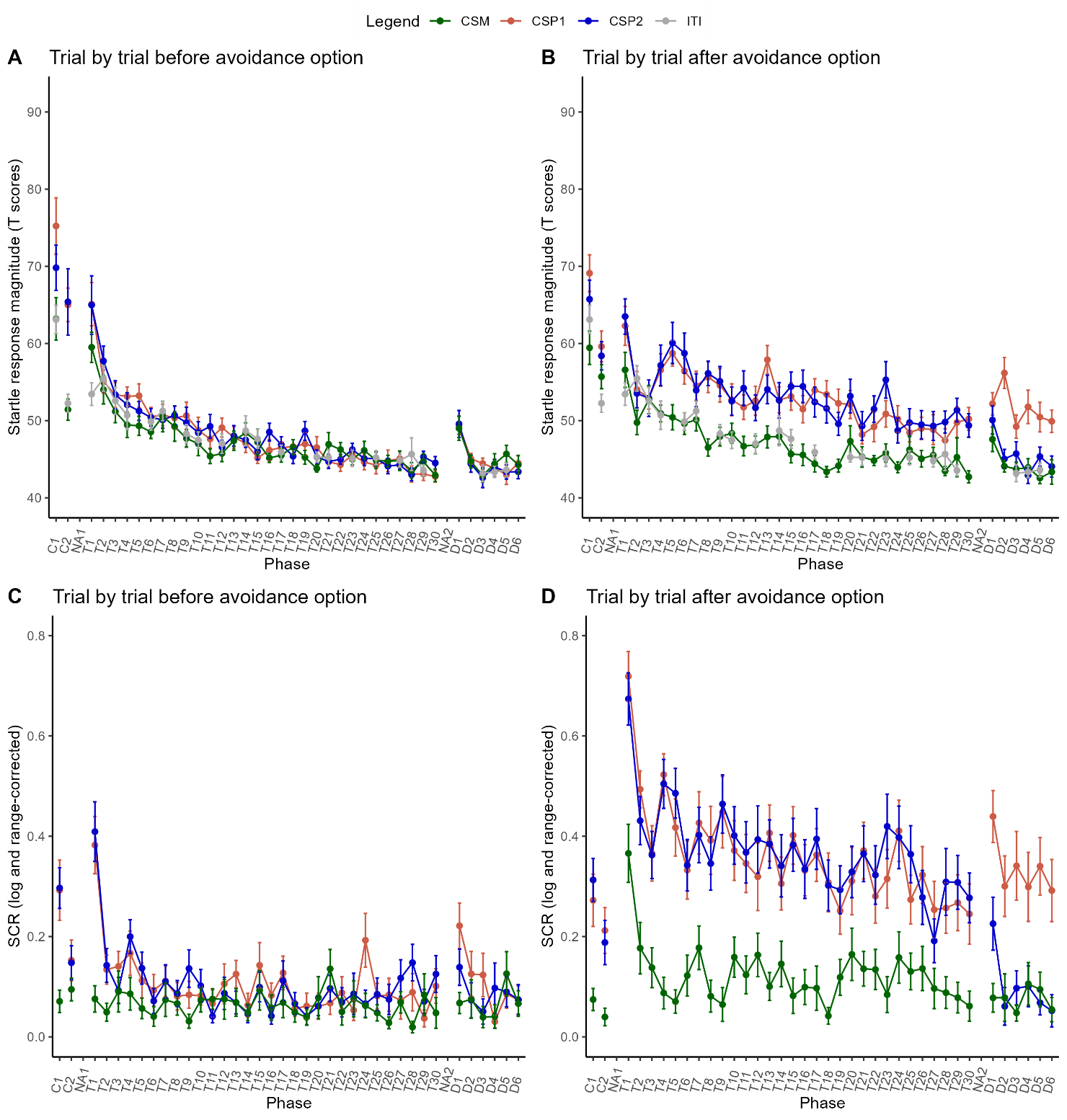


*Note.* Plots on a trial-by-trial basis (x-axis) for startle response magnitude (top panel) and skin conductance response (bottom panel) before and after the avoidance option.

1. [↑](#footnote-ref-1)
